# Daily Practice Managing Resistant Multiple Sclerosis Spasticity With Delta-9-Tetrahydrocannabinol: Cannabidiol Oromucosal Spray: A Systematic Review of Observational Studies

**DOI:** 10.1177/1179573519831997

**Published:** 2019-03-11

**Authors:** Katja Akgün, Ute Essner, Cordula Seydel, Tjalf Ziemssen

**Affiliations:** 1 Department of Neurology, Center of Clinical Neuroscience, University Hospital Dresden, Dresden, Germany; 2 O.MEANY Consultancy GmbH, Hamburg, Germany; 3 Almirall Hermal GmbH, Reinbek, Germany

**Keywords:** Central nervous system, multiple sclerosis, spasticity, cannabinoids, real-world data

## Abstract

**Background/purpose:**

Spasticity is one of the most common symptoms in people with multiple sclerosis (MS). Conventional anti-spasticity agents have limitations in their efficacy and tolerability. Delta-9-tetrahydrocannabinol: cannabidiol (THC:CBD) spray, a cannabinoid-based medicine, is approved as an add-on therapy for MS spasticity not adequately controlled by other anti-spasticity medications. The results from randomized controlled trials (RCTs) have demonstrated a reduction in the severity of spasticity and associated symptoms. However, RCTs do not always reflect real-life outcomes. We systematically reviewed the complementary evidence from non-interventional real-world studies.

**Methods:**

A systematic literature review was conducted to identify all non-RCT publications on THC:CBD spray between 2011 and 2017. Data on study design, patient characteristics, effectiveness, and safety outcomes were extracted from those publications meeting our inclusion criteria.

**Results:**

In total, we reviewed 14 real-world publications including observational studies and treatment registries. The proportion of patients reaching the threshold of minimal clinical important difference (MCID), with at least a 20% reduction of the spasticity Numeric Rating Scale (NRS) score after 4 weeks ranged from 41.9% to 82.9%. The reduction in the mean NRS spasticity score after 4 weeks was maintained over 6-12 months. The average daily dose was five to six sprays. Delta-9-tetrahydrocannabinol: cannabidiol was well tolerated in the evaluated studies in the same way as in the RCTs. No new or unexpected adverse events or safety signals were reported in everyday clinical practice.

**Conclusions:**

The data evaluated in this systematic review provide evidence for the efficacy and safety of THC:CBD in clinical practice and confirm results obtained in RCTs.

## Introduction

Multiple sclerosis (MS) is a progressive, long-term autoimmune demyelinating disease of the central nervous system.^1^ A variety of symptoms are commonly associated with MS, spasticity being the most frequent one.^2–4^ Spasticity may occur in around 80% of patients within the first decade after MS diagnosis in severity over time.^3,5,6^

Features of MS spasticity comprise increased muscle tone during active movements or passive stretching, unprovoked persistent raised muscle tone, and/or transient painful paroxysmal muscle spasms.^7^ Further symptoms commonly associated with MS spasticity apart from spasms are sleep disturbances, pain, fatigue worsening, and bladder dysfunction.^4,8^ The worsening of mobility due to spasticity has a negative impact on quality of life (QoL) in MS patients and contributes to disability. Severity of MS spasticity directly correlates with the degree of impairment of daily activities.^5,8–12^ If spasticity is not treated, secondary physical and functional complications may arise.^13^

Although different methods are available to assess the degree of spasticity in MS patients, spasticity is often not documented in a standardized way in neurology services clinical practice. Among available scores, the (Modified) Ashworth Scale (MAS) is the most widely used physician-rated tool.^14^ Other patient-rated scales include the Visual Analog Scale (VAS), the Numeric Rating Scale (NRS),^15^ or the Multiple Sclerosis Spasticity Scale (MSSS-88) for measuring the impact of spasticity on QoL.^16^ Apart from spasticity itself, these scales can evaluate different symptoms associated with spasticity such as stiffness, clonus, spasms, pain, and overall comfort.^17^ The range of instruments for the clinical measurement of spasticity is limited, and no single instrument is valid for all cases. Thus, assessing the effects of anti-spasticity treatment may require different outcome measures typically not gathered in clinical practice to obtain meaningful results.

Treatment options for MS spasticity include physiotherapy and/or anti-spasticity agents such as baclofen, dantrolene, tizanidine, gabapentin/pregabalin^18^ as well as cannabis-based medications. All of these have different modes of action, efficacy, and tolerability profiles, influencing their role in symptomatic MS treatment.^19^ Many patients fail to respond sufficiently to classical treatments and/or suffer from adverse reactions especially with prolonged use and high dosages.

For these patients, delta-9-tetrahydrocannabinol: cannabidiol (THC:CBD) oromucosal spray (Sativex^®^), may be an alternative therapeutic option. Delta-9-tetrahydrocannabinol: cannabidiol spray is a cannabinoid medicine derived from *Cannabis sativa* plants. It has been approved as an add-on therapy “for symptom improvement in adult patients with moderate to severe spasticity due to multiple sclerosis (MS) who have not responded adequately to other anti-spasticity medication and who demonstrate clinically significant improvement in spasticity related symptoms during an initial trial of therapy.” Application is via the oromucosal route. The spray contains two active substances in a ratio of 1:1, delta-9-tetrahydrocannabinol (THC) and Cannabidiol (CBD). Both compounds are thought to possess complementary properties. Delta-9-tetrahydrocannabinol acts on cannabinoid receptors CB1 and CB2 as a partial agonist and can modulate the excitatory effects of glutamate and the inhibitory effects of gamma-aminobutyric acid (GABA), achieving muscle relaxation and improvement of spasticity. It is believed that CBD, as it acts as an antagonist at CB2 receptors, can reduce some unwanted reactions to THC such psychoactive effects.^20^

THC:CBD spray was granted marketing authorization in Canada in 2005 and in a growing number of European Union (EU) countries and other areas since 2010. Clinical experience with THC:CBD spray has accumulated in over 20 countries since its launch, and global exposure is estimated to exceed 55 000 patient-years by the end of 2016. Findings from several randomized controlled trials (RCTs) have consistently shown that THC:CBD spray significantly reduces symptoms of MS spasticity.^21-24^ These results were confirmed in long-term studies.^25,26^ Earlier clinical studies with THC:CBD had shown that intention-to-treat analysis was underestimating the efficacy in a patient population with a high proportion of non-responders, that is, in patients with moderate to severe MS spasticity not adequately responding to or not tolerating conventional anti-spastic drugs (eg baclofen and tizanidine). To overcome the underestimation of the efficacy, later studies used an enriched study design starting with a single-blind (all subjects allocated to treatment) 4-week treatment period to identify patients with an initial treatment response. Those with at least a 20% reduction in mean NRS spasticity score within the first 4 weeks were classified as initial responders (IRs) suitable for continued therapy.^23,24^

RCTs with their robust methodology are considered the gold standard for obtaining clinical data on efficacy and safety of therapeutic interventions. However, they may miss relevant data, as interventions are evaluated in a protocol-driven, ideal experimental setting. In RCTs, patients are selected to meet certain inclusion and exclusion criteria according the study protocol such as age, co-morbidities, and co-medication. The generalisability and applicability of the RCT results to everyday clinical practice is therefore limited. Collecting data on treatment in real-world clinical practice can bridge this gap. Real-world data are increasingly regarded as complementary sources of data to RCTs, as they may include a more diverse group of patients in routine clinical practice and provide long-term results on outcomes and compliance.^27^ Real-world data can be obtained from a variety of sources and research methodologies which include databases, registries, medical record reviews, prospective or retrospective patient data collections, case series, or classic real-world studies such as observational cohort studies.^28^ All of these data sources usually have different aims and limitations. They may not be complete or representative. Retrospective data on the one hand may contain patients not treated or analyzable and prospective data on the other hand may be biased and confounding.^29^ By combining and evaluating data from various real-world sources, these limitations can be overcome and valid real-world evidence obtained.^27^

The aim of our systematic review was to evaluate the real-world evidence for the benefits and safety of THC:CBD spray in the symptomatic treatment of refractory spasticity due to MS as it has been done for other MS drugs before.^30^ We planned to include a variety of medium- to high-quality real-world literature sources.

## Methods

### Study design and search strategy

We conducted a systematic literature review according to a pre-specified protocol including a systematic literature search, study selection, and subsequent data extraction. Based on a comprehensive search strategy, an electronic literature search was performed to identify publications reporting non-interventional (observational) studies and registry data on the use of THC:CBD oromucosal spray in the treatment of MS-related spasticity since its commercial availability in EU countries in 2011 to October 2017. We searched Medline, Embase, and the Cochrane library. In addition, the bibliographies of the included publications and of any meta-analyses and systematic reviews were hand searched to identify further relevant publications. The complete list of search keywords is shown in Table A1 of Appendix 1.

### Study selection and data extraction

The search results from different databases were combined and duplicates removed. All remaining study titles and abstracts were filtered by two independent reviewers. Publications meeting the inclusion criteria and none of the exclusion criteria were obtained as full text and reassessed for eligibility (Table 1).

**Table 1.** Eligibility criteria used in the evaluation of studies.

	Inclusion	Exclusion
Population	Adult human patients with multiple sclerosis	Animal/in vitro studies, Pediatric patients
Interventions	Sativex post-marketing authorization	Studies not including Sativex
Study design	Observational, non-randomized, non-interventional Cohort studies Case-control studies Registry studies Before-and-after studies Prospective/retrospective studies Questionnaires Longitudinal, follow-up studies	General reviews, systemic reviews, meta-analyses Congress abstracts RCTs Preclinical, phase 1 studies Pilot data Case reports, case series reports Pharmacodynamic studies Pooled, post hoc, secondary analyses Economic evaluations Editorials, commentaries
Outcome	Patient-relevant outcomes, for example, symptoms of spasticity, spasticity-related symptoms, functional status Activities of Daily Living Quality of life Safety Discontinuation	Costs Cost-effectiveness Pain

Randomized controlled studies, preclinical studies, pilot data, case reports, conference abstracts, and secondary analyses were excluded.

The full texts of the published papers were scrutinized by the authors. Data were extracted using a standardized data extraction form (Table 2) to compile details of the following parameters:

- Study design.
- Study size.
- Population (setting and locations).
- Defined outcome parameters.
- Follow-up period.
- Limitations.

**Table 2.** Summary of included real-life clinical trials.

Author	Study design and aim	Country	Study cohort	MS parameters at baseline	Indication for use of Sativex	Length of follow-up	Mean dose (sprays/day)	Outcome measures	Clinical outcome	Safety
Etges et al^31^	Retrospective post-marketing safety registry to collect long-term data	UK, Germany, Switzerland	N = 941 (57% female) Age 51.2 (SD ± 10.8)	Disease duration: NR EDSS: NR	59% confirmed MS spasticity as per approved label; 13% off-label	4.5 years (maximum) 2-3 months data collection periods	5.4 (SD ± 4.9)	Benefit (no parameters specified) Special interest long-term AEs (falls, psychiatric, suicidal thoughts, and driving ability)	NRS: NR 60% continued treatment throughout the observation period 83% reported benefit in at least one observation periods 32% had discontinued treatment due to the lack of effectiveness, AEs and others	13.1% of patients with ⩾1 treatment-related AE, of which: 2.9% psychiatric; 2.3% dizziness; 1.7% fatigue; 3.4% gastrointestinal No indication for abuse, dependence, or new long-term safety concerns
*Patti^32^	Prospective registry of all Italian patients prescribed Sativex (AIFA), figures after 1.5 years	Italy	N = 1534 (52.8% female); Age 51 (SD ± 9.6)	Disease duration: 17.6 years (SD ± 8.6) EDSS: 6.4 (SD ± 1.2)	MS spasticity as per approved label	6 months Assessment at baseline, 1 month, 6 months	6.2 (SD ± 2.8) at 6 months	NRS (spasticity) at baseline, month 1 and 6 Discontinuation	NRS baseline: 7.6 (SD ± 1.4) NRS 1 month: 5.3 (SD ± 1.3); NRS 6 months: NR 70.3% ⩾20% NRS response after 1 month; 39% continued treatment at 6 months 29.7% had discontinued at 3 months due to lack of effectiveness (15%), AEs (9.3%) etc. 55.3% had discontinued at 6 months	15.9% of patients with ⩾1 treatment-related AE, of which: 2.8% cognitive/psychiatric; 2.7% fatigue; 2.0% dizziness; 1.4% gastrointestinal; 0.7% oral discomfort AEs mainly mild to moderate. No new safety concerns
Patti et al^33^ (continuation of Patti^32^)	Prospective registry of all Italian patients prescribed Sativex (AIFA)	Italy	N = 1615; (52.6% female); Age 51 (SD ± 9.5)	Disease duration: 17.5 years (SD ± 8.6) EDSS: 6.5 (1.5-9.5)	MS spasticity as per approved label	6 months Assessment at baseline, 4 weeks, 3 and 6 months	6.3 (SD ± 2.8) at 6 months	NRS (spasticity) at baseline, month 1, 3, and 6 Discontinuation	NRS baseline: 7.4 (SD ± 1.5) NRS 1 month: 5.9 (SD ± 1.6) NRS 3 months: 5.1 (±1.6) NRS 6 months: 4.8 (±1.7) 70.5% ⩾20% NRS response after 1 month 55.6% continued treatment at 3 months 36.7% continued treatment at 6 months 39.5% had discontinued at 6 months due to lack of effectiveness (23.2%) and/ or AEs (16.3%)	% with ⩾1 treatment-related AE: NR 3.2% psychiatric; 2.5% fatigue; 2.0% dizziness; 1.4% gastrointestinal; 0.7% oral discomfort AEs in line with other observational studies. No abuse, addiction, and misuse
Messina et al^34^	Prospective, observational registry (AIFA) to describe Sativex discontinuation profile (see also Patti^32,33^)	Italy	N = 1597 (53% female) Age 51 (21-84)	Disease duration: 17.5 years (SD ± 8.6) EDSS: 6.5 (1.5-9.5)	MS spasticity as per approved label	Median follow-up 730 days (2 years) (range: 2-730) Assessment at baseline, 4 weeks, 3 and 6 months	6.5 (SD ± 2.6) at 3 months, 6.3 (SD ± 2.8) at 6 months	NRS (spasticity) Discontinuation	NRS baseline: 7.5 (SD ± 1.4) NRS 1 month: 5.9 (SD ± 1.6) NRS 3 months: 5.1 (SD ± 1.6) NRS 6 months: 4.8 (SD ± 1.7) 24.8% had no ⩾20% NRS response after 1 month 39.5% discontinued throughout observation period (20.8% within 4 weeks) due to lack off effectiveness (23.2.%), AEs (16.3%), non-adherence etc.	AE-details in previous paper by Patti^32,33^
Vermersch and Trojano^35^	Prospective observational multicentre study to determine the overall effectiveness and tolerability/safety in everyday clinical practice MOVE-2 EU	Europe (Germany, Italy, Norway, Denmark)	N = 433 (432 in analysis) (55.2% female) Age 50.4 (SD ± 10.4)	Disease duration: 13.7 years (SD ± 7.9) EDSS: 5.94 (SD ± 1.38)	MS spasticity as per approved label	3 months Assessment at baseline, 1 and 3 months	5.7 at 3 months	ADL; MAS; NRS (spasticity, sleep impairment, fatigue, and pain); QOL; Likert-type Scales (spasticity, pain, and fatigue); number of spasms per day, night awakenings, urinary incontinence per week	NRS baseline 6.9 (±1.9) NRS 1 month: NR NRS 3 months: 5.3.(±1.8) 80.6% ⩾20% NRS response after 1 month 65% continued treatment at 3 months 18.5% had discontinued therapy at 3 months due to lack of effectiveness, AEs	10.4% of patients with ⩾1 treatment-related AE, of which: 2.1% psychiatric; 0.7% fatigue; 3.7% dizziness; 1.4% gastrointestinal AEs in line with the known safety profile
Trojano and Vila^36^	Prospective observational, multicentre study (MOVE-2 EU) to determine the overall effectiveness and tolerability/ safety in everyday clinical practice MOVE-2 EU Interim analysis of Italian data after 3 months	Italy	N = 322 (of which 295 in analysis) (58.3% female) Age 51.1 (SD ± 10.2)	Disease duration: NR EDSS: NR	MS spasticity as per approved label	3 months (interim analysis) Assessment at baseline, 1 and 3 months	6.1 (SD ± 2.5) at 1 month, 5.1 (SD ± 2.6) at 3 months	ADL; MAS; NRS (spasticity, sleep impairment, fatigue, and pain); QOL; Likert-type Scales (spasticity, pain, and fatigue)	NRS baseline 6.8 (SD ± 1.9) NRS 1 month: NR NRS 3 months: 5.5 (SD ± 1.6) 82.9% ⩾20% NRS response after 1 month MAS baseline: 2.6 (SD ± 0.8) MAS 1 month: 2.2 (SD ± 0.8) 8.7% had discontinued at 1 month 14% had discontinued at 3 months due to lack of effectiveness, lack of tolerability etc.	13.1% of patients with ⩾1 treatment-related AE, of which: dizziness (5.6%); confusion (2.5%); somnolence (1.25%); nausea (1.25%) AEs in line with the known safety profile
Flachenecker et al^37^	Prospective observational multi-center study to evaluate the effectiveness and safety of nabiximols in everyday clinical practice MOVE-2	Germany	N = 300 (of which 276 in analysis) (60.9% female) Age 50.0 (SD ± 9.4)	Disease duration: 15.4 years (SD ± 9) EDSS: 6.0 (1.0-9.0)	MS spasticity as per approved label	3-4 months Assessment at baseline, 4 ± 2 and 12 ± 4 weeks	6.9 (SD ± 1.38) at one month; 6.7 (SD ± 2.9) at 3 months	NRS (spasticity, sleep disturbance); ADL; MAS; QoL	NRS baseline 6.1 (SD ± 1.7) NRS 1 month: 5.2 (SD ± 1.9) NRS 3 months: 4.7 (SD ± 2.0) 41.9% ⩾20% NRS response after 1 month MAS baseline: 3.0 (±0.8) MAS 1 month: 2.7 (±0.9) 79.0% continued at 1 month 55.3% continued at 3 months 36.3% had discontinued at 1 month due to lack of effectiveness, AEs, etc.	15.7% of patients with ⩾1 treatment-related AE, of which: 2.5% fatigue; 4.0% dizziness; 1.9% nausea No new safety concerns. Safety profile similar to clinical studies
Flachenecker et al^38^**	Prospective observational multicentre study to evaluate the effectiveness and safety of nabiximols in everyday clinical practice Prolongation of the 3 month MOVE-2^25^	Germany	N = 104 entered prolongation, of which 52 with 12-month follow-up (55.8% female) Age 49.4 (SD ± 8.6)	Disease duration: 14.1 years (SD ± 8.0) EDSS: 6.0 (3-8)	MS spasticity as per approved label	12 months	6.2 (SD ± 2.6) at 12 months	NRS (spasticity, sleep disturbance); ADL; MAS; QoL	NRS baseline 6.2 (SD ± 1.8) NRS 12 months: 4.6 (SD ± 2.1) 52.9% ⩾20% NRS response after 1 month 62% continued at 12 months 29.8% had discontinued due to lack of effectiveness (9.6%), AEs (4.8%) etc.	16.3% of patients with ⩾1 treatment-related AE, of which: 3.9% psychiatric; 1.0% fatigue; 1.0% dizziness; 5.8% gastrointestinal No new safety concerns Overall AE profile similar to that of clinical studies
Ferre et al^39^	Prospective observational monocentric study to investigate the efficacy and safety of nabiximols in a real-world cohort	Italy	N = 144 (52.2% female) Age 49.7 (SD ± 10.3)	Disease duration: 17.6 years (SD ± 8.5) EDSS: 6.5 (2.0-8.5)	MS spasticity as per approved label	1 year Assessment at baseline, 4, 14 and 48 weeks	6.4 (SD ± 2.3) at 14 weeks	NRS (spasticity, pain); MAS; 10 MWT	NRS baseline: 7.5 (SD ± 1.3) NRS 1 month (in responders): 5.2 (SD ± 1.2) 71.7% ⩾20% NRS response after 1 month 62.5% continued at 14 weeks 35% had discontinued therapy at 14 weeks due to lack of effectiveness, AEs (13.9%) etc.	80.5% with ⩾1 AE (all AEs, no differentiation of treatment-related AEs) Dizziness, confusion, fatigue being most common and mainly occurring during titration phase Data in agreement with RCTs. Collected all possible AEs which occurred at least once. Usually mild and transient
Paolicelli et al^40^	Prospective observational, monocentric study on efficacy, tolerability, safety	Italy	N = 102 (49% female) Age 48.8 (SD ± 10.4)	Disease duration: 19.2 years (SD ± 8) EDSS: 6.7 (SD ± 1.1)	MS spasticity as per approved label	40 weeks (SD ± 28 weeks) Assessment at baseline, 4 weeks, 3,6 and 12 months	6.5 (SD ± 1.6)	NRS (spasticity); Ambulation Index (AI); Timed 25-Foot Walk (T25-FW); Patient global impression of change (PGIC)	NRS baseline: 8.7 (SD ± 1.3) NRS 1 month: 6.2 (SD ± 1.8) NRS 3 months: 5.9 (SD ± 1.6) NRS 6 months: 6.1 (SD ± 1.4) NRS 12 months: 6.3 (SD ± 1.4) ⩾20% NRS response after 1 month: NR 36.2% discontinued throughout the observation period due to lack of effectiveness (17.7%), persistent AEs	40.2% incidence of AEs, most common dizziness, psychiatric, and gastrointestinal Overall incidence of AEs slightly lower than in pivotal study (Novotna) possibly due to lower daily doses used
Oreja-Guevara et al^41^	Prospective observational multi-centre safety study	Spain	N = 205 (62% female) Age 48.6 (SD ± 9.7)	Disease duration: NR EDSS: NR	MS spasticity as per approved label	12 months Assessment at baseline, 6 and 12 months	6.6 (SD ± 2.8) at 12 months	Safety analysis (addiction, misuse, mood changes, memory impairment, driving ability, and falls)	NRS: NR 68% continued at 6 months 60.5% continued at 12 months with sufficient clinical benefit 35.6% had discontinued due to AEs and/or lack of effectiveness	20% with ⩾1 AE (all AEs) 2/3 of the treatment-related AEs Most common gastrointestinal (diarrhea, oral mucosa), psychiatric, and dizziness No new safety signals compared to RCTs and no clinically relevant AEs of special interest (such as falls, psychiatric symptoms, memory impairment, addiction, abuse, changes in driving ability)
Lorente Fernandez et al^42^	Retrospective observational, monocentric study to evaluate effectiveness and safety in clinical practice	Spain	N = 50 (58% female) Age 47.8 (25.6-76.8)	Disease duration: NR EDSS: NR	MS spasticity as per approved label and/or pain	Median exposure time: 30 (5-263) days in those who discontinued: 174 (23-1422) days in those who continued	5 (2-10)	Response (dichotomous, that is, yes/no answer based on prescriber’s analysis and impression); exposure time	NRS: NR In 80% of patients response 32% discontinued due to lack of effect (14%), AEs (10%), non-compliance (8%)	52% of patients with ⩾1 treatment-related AE, of which: dizziness; muscle weakness; somnolence and oral discomfort, and diarrhea are the most common Incidence of AEs lower than in meta-analysis by Sastre-Garriga but similar to RCT (Novotna)
Koehler et al^43^	Retrospective observational, monocentric study Medical chart data collection	Germany	N = 166 (58% female) Age: 49 (mean)	Disease duration: NR EDSS in RRMS: 6.0 (SD ± 1.7) SPMS: 7.5 (SD ± 1.0) PPMS: 7.5 (SD ± 1.0)	MS spasticity as per approved label; (mono-therapy in 20.8% due to intolerance of other anti-spasticity therapy)	15 months (9-15 months treatment)	4 (±2.6)	NRS (spasticity)	NRS (baseline, responder): 7.0 (4-10) NRS (10 days): 3.0 (0-6) 27.7% discontinued during observation period due to AEs (13.9%), lack of efficacy (8.4%) or need for baclofen pump (5.5%)	13.9% of patients with ⩾1 treatment-related AE leading to discontinuation of which dizziness (3%), fatigue (3%), oral discomfort (2.4%) most common No new safety signals
Freidel et al^44^	Prospective observational, multicentre study to determine effects on driving ability	Germany	N = 33 (31 completed) (60.6% female) Age: 48.1 (mean; 33-68)	Disease duration: 11.5 years (SD ± 6.8) EDSS: 4.6	MS spasticity as per approved label	5.3 weeks (mean: 4-6)	5.1 (mean)	Driving ability tests (2 approaches): Strict: if a percentile rank of at least 16% achieved in each of the five subscores Modified: if a score below 16% in one or more tests was compensated by a ⩾50% In another test NRS (spasticity)	NRS baseline: 6.0 (median) NRS 4-6 weeks: 3.0 (median) 6% discontinued prematurely due to lack of effectiveness (3%) and AEs (3%)	9.1% (3/33) of patients with ⩾1 treatment-related AE of which dizziness (6.1%) and vertigo (3.0%) 45.2% “fit to drive” under strict criteria. 77.8% “fit to drive” under modified criteria 6.5% changed categories from “fit” at baseline to “unfit” at final visit and vice versa, that is, no change of frequencies of patients classified as “fit to drive”

Abbreviations: ADL, Activities of Daily Living scale (Barthel index); AE, adverse event; EDSS, Expanded Disability Status Scale; MAS, Modified Ashworth Scale; MS, multiple sclerosis; MWT, time to walk 10 meters; QoL, quality of life; NRS, Numerical Rating Scale; NR, not reported; RRMS, relapsing-remitting multiple sclerosis; PPMS, primary progressive multiple sclerosis; SPMS, secondary progressive multiple sclerosis.

* The same registry, analyzed at different time points; **long-term follow-up of MOVE-2.^37^

The review was fully consistent with the 2009 Preferred Reporting Items for Systematic Reviews and Meta-Analyses (PRISMA) guidelines.^45^

The following criteria based on the proposed standards for real-world evidence by Ziemssen et al^46^ were used to assess the quality of the selected studies: defined inclusion/exclusion criteria, representative sample, that is, multi-center, defined outcomes according to objective criteria, sufficient follow-up period long enough to assess outcome, and sources of bias/confounding identified.

## Results

### Search yields

The electronic literature searches were performed in Medline (PubMed) on October 04 2017, in Embase on August 16 2017, and in the Cochrane Library on October 04 2017.

**Figure 1.**
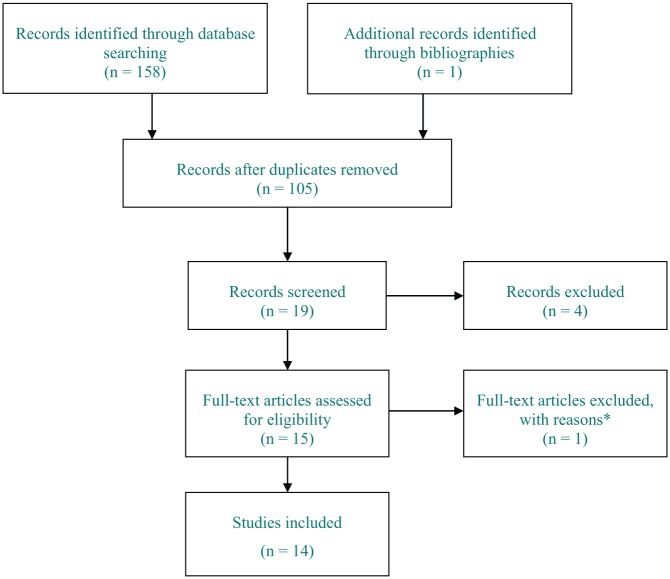
PRISMA flow chart of included and excluded studies. *Full-text article was a review.

The procedure and outcome of the electronic search is summarized in Figure 1. After the assessment of titles, abstracts, and full papers, 14 publications were included in the review.

### Properties of included published papers

Table 2 summarizes the main characteristics and results of the 14 finally included observational studies investigating the THC:CBD spray. In total, four of the publications present registry databases; three of them analyzing the same sample from the Italian Medicines Agency (AIFA) web-registry which is mandatory for the follow-up of all patients receiving Sativex in Italy^32–34^ and the fourth reporting data on a multi-center observational safety registry opened in the United Kingdom, Germany, and Switzerland as part of the European Medicines Agency (EMA)-requested risk management plan to monitor potential safety signals not detectable by short-term RCTs.^31^ The remaining publications report non-interventional studies (NISs) of observational nature (n = 10). Most of the publications were prospective (n = 11), whereas three publications were described by the authors as retrospective (n = 3).

Multi-center, prospective observational studies we included, were the MOVE-2 Italy EU study (albeit had a few cases coming from Norway too) with interim results published by Trojano and Vila^36^ and final results by Vermersch and Trojano^35^ as well as the first MOVE-2 equivalent protocol study conducted in Germany by Flachenecker et al^37^ with a follow-up period of 3 months and with long-term data at 12 months,^38,41^ a Spanish long-term follow-up safety study by Oreja-Guevara et al^41^ and a monocentric medical chart data collection by Koehler et al.^43^ We also identified smaller monocentric studies such as two Italian prospective studies with long-term data over 40-48 weeks,^39,40^ a retrospective observational study by Lorente Fernandez,^42^ and a prospective NIS evaluating the effects on driving ability by Freidel et al^44^ (Table 3).

**Table 3.** Summary of the characteristics of the included real-world publications.

	Number of studies (n)	References
Study design (as stated by the author)		
Prospective	7	33,35,37,39-41,44
Retrospective	3	31,42,43
Data source		
Non-interventional studies	8	
Multicenter	4	37,38 (Move-2, Move-2 long-term),^35^,^36^ (Move-2 EU, evaluation at two different time points), 41,44
Single center	4	39,40,42,43
Registries	2	
AIFA (Italy)	3	32-34 (evaluation at three different time points)
Safety registry (United Kingdom, Germany, Switzerland)	1	31

Abbreviations: EU, European Union; AIFA, Italian Medicines Agency.

Table 4 demonstrates the results of the quality assessment of all included studies. The quality of most of the studies was considered high, as they met all of the applied standards. Three studies, two of which were monocentric and one multi-center study investigating the potential impact on driving ability lacked two or more quality criteria. They were identified as medium quality.^42-44^ Further medium quality sources comprise the MOVE-2 EU study with a short follow-up period of only 3 months and the international safety registry where approved use of THC:CBD was not confirmed in all patients.^31,35,36^

**Table 4.** Quality assessment of the included publications.

Author	Defined inclusion/exclusion criteria?	Representative sample from the relevant population, that is, multicenter?	Defined outcome according to objective criteria?	Follow-up period long enough to assess outcome?	Statistical analyses and sources of bias/confounding identified?
Etges et al^31^	No inclusion criteria apart from prescription of THC/CBD	Yes, multicentre, international	No (long-term safety, special interest AEs)	Yes	Only one safety analysis set
Patti et al^32,33^	Yes, patients treated according to approved label	Yes, multicentre, Italy	Yes	Yes	Yes, statistical analysis performed
Messina et al^34^	Yes, patients treated according to approved label	Yes, multicentre, Italy	Yes	Yes, median 730 days (2 years)	Yes, statistical analysis performed
Vermersch and Trojano^35^ and Trojano and Vila^36^	Yes	Yes, multicentre, international (MOVE-2 EU)	Yes	No, 3 months	Yes, statistical analysis performed (effectiveness on full analysis set)
Flachenecker et al^37,38^	Yes	Yes, multicentre	Yes	Yes as prolongation 12 months	Yes, descriptive statistical methods
Ferre et al^39^	Yes	Yes, monocentric, but sufficient number of patients	Yes	Yes	Yes, descriptive statistical methods
Paolicelli et al^40^	None specified	Yes, monocentric, but sufficient number of patients	Yes	Yes	Yes, statistical analysis performed
Oreja-Guevara et al^41^	Yes	Yes, multicenter	Yes (safety analysis, special interest AEs)	Yes	Yes, statistical safety analysis (descriptive statistical methods for baseline characteristics)
Lorente Fernandez et al^42^	No	No, monocentric, N = 50	No	Yes	Yes, statistical analysis performed
Koehler et al^43^	No	Yes, monocentric, but sufficient number of patients	Yes	Yes	No statistical analysis
Freidel et al^44^	Yes	No, multicentre, but N = 33	Yes	No	Yes, statistical analysis performed

Abbreviations: AE, adverse events; THC:CBD, delta-9-tetrahydrocannabinol: cannabidiol.

### Study designs

Patients in the observational studies on THC:CBD were treated in accordance with the approved label except for the multinational safety registry, in which the therapeutic indication of MS spasticity was not confirmed in all cases,^31^ and one monocentric study, wherein 10% of MS patients were treated for pain.^42^ Continued use of additional anti-spasticity medication as per approved label recommendations was reported in most studies. Monotherapy with THC:CBD was described in 20% of the patients in one of the monocentric studies because of intolerance of other anti-spasticity therapies.^43^

The main focus of the registries was to gather safety data and information on dosages used in everyday clinical practice^31,41^ with the exception of the mandatory e-registry of the AIFA which also documented effectiveness data.^32,33^

In some of the registries and one of the NIS, prescribers were asked to provide data on special interest safety events (risk of falls, suicidality, psychosis, abuse liability, and effect on driving ability) besides the usual documentation of adverse and serious adverse effects.^31,41^ The data collected in the NIS typically comprised effectiveness and safety parameters. Some of the studies assessed additional effectiveness outcomes such as associated symptoms (pain, sleep interruptions, and bladder dysfunction), QoL, and impairment in activities of daily living (ADL; Table 2)^35–38^ and/or additional safety outcomes such as driving ability.^44^

### Demographic data and baseline characteristics

The total number of patients in our review is 3989, female being the more common gender (mean, 56%). It is worth noting that we included a few publications analyzing data of the same study or registry at different time points. The largest data collection, the AIFA registry, had recruited 1615 patients at the last published analysis.^33^ In contrast, 31 patients completed the smallest NIS focusing on driving ability.^44^ On a non-weighted average, patients were 50 (range: 48-52) years. The youngest patients enrolled were 18 years of age, the oldest 85 years.^31^

The mean MS disease duration was 16 years ranging from 6.7^44^ to 19.2 years.^40^ None of the studies excluded patients on the basis of MS subtype. The disability of the MS patients in most of the NIS and registries was assessed by means of the Expanded Disability Status Scale (EDSS), as its levels have been shown to be related to spasticity.^9^ The mean EDSS score was 6.2, representing ambulation disability.^47^

### Drug exposure and dose

The duration of drug exposure differed in the registries and NISs (Table 2). The follow-up period in the registries ranged from 6^32,34^ to 12 months,^41^ with the longest exposure time being 4.5 years in the international safety registry.^31^ In the NIS, the follow-up duration varied from 1 month^44^ to 4 years.^42^ The mean recorded daily dose of THC:CBD in the publications was in the range of four to seven sprays.

### Spasticity outcome

In most of the NIS collecting effectiveness data, the outcome for MS spasticity was assessed by the 0-10 MS spasticity NRS change and additionally in some studies by the MAS. The enriched trial design from RCTs to identify IRs with at least a 20% reduction in mean NRS spasticity score within the first 4 weeks was adopted in most of the real-world studies.^23^ The proportion of IRs varied between 41.9%^37^ and 82.9%.^36^ In the AIFA registry, which assessed the largest number of patients, an initial response was seen in 70.5% of patients. Furthermore, up to 28% of the patients had reached a clinical relevant response (CRR), that is, a ⩾30% reduction in MS spasticity NRS after 4 weeks of treatment.^8,33^

Some studies assessed ambulatory function using the time needed to walk 25 feet (25-Foot Walk = T25-FW)^40^or 10 m (10MWT)^39^ or the Ambulation Index (AI) scale on time and degree of assistance required to walk 25 feet (8 m).^40^ During THC:CBD treatment, an improvement in the 10MWT was observed in responders with a reduction from 25.5 seconds (SD ± 18.9) at baseline to 21.6 seconds (SD ± 13.8) after 1 month (*P* < .001).^39^ This was in line with a significant decrease in the AI score after 1 month.^39^ Likewise, the T25-FW test performed in another monocentric study significantly improved in comparison to baseline within the first month.^40^

Long-term real-world data showed that the reduction in the mean NRS spasticity score after 4 weeks was maintained over 6-12 months.^33,38,40^ A reduction in spasticity of more than 30% (CRR) was shown in 35%-40% of the patients after 3 months,^33,37^ in 43% after 6 months,^33^ and in 40% after 12 months of treatment.^33,37^ Those studies additionally assessing spasticity using MAS also showed a significant decrease after 1 month compared to baseline.^36,37^

### Spasticity-associated symptoms

In the observational studies examining the effects of THC:CBD on secondary outcomes, the improvements in spasticity outcomes corresponded with significant improvements in associated symptoms. In the German MOVE-2 study, the mean NRS score for sleep disturbances decreased by 24.3% within the first month of treatment.^37^ Moreover, a statistically significant reduction was observed within the first month of treatment in the number of patients who considered muscle stiffness, restricted mobility, pain, and bladder disorders as their most disturbing symptoms.^37^ In the MOVE-2 Italy study, significant improvements in most of associated symptoms including spasms counts, sleep impairment, number of night awakenings caused by spasticity, fatigue, pain, and the number of urinary incontinence episodes per week were reported at 3 months.^35^

### Discontinuation

Around 30%-39% of patients in the large registries permanently discontinued THC:CBD throughout the observation periods.^31,33,34^ Similar percentages of discontinuation rates were seen in the NIS^38–40^ with the exception of the MOVE-2 Italy study with only 18.5% of patients who had stopped THC:CBD after 3 months.^35^ Reasons stated for discontinuation were consistently either the lack of effectiveness and/or adverse events (AEs).

### Quality of life and activities of daily living

Quality of life was examined in the MOVE-2 studies. In terms of MS-specific quality of life (MSQoL-54), statistically significant improvements of the physical health composite score were seen over a 3-month period in the MOVE-2 Study in Germany.^37^ In the MOVE-2 Italy study, the patient-reported QoL showed no significant improvements in the 5 EQ-5D categories, while the mean (median) score for the overall state of health assessed by the EQ 0-100 VAS improved significantly from baseline to month 3.^35^

The impairment of daily activities was measured in patients enrolled in the MOVE-2 studies using Barthel Index. In line with the relief of MS spasticity, the ADLs improved: the number of patients with restrictions in many daily activities decreased significantly from 30.2% at baseline to 22.8% after 3 months in the MOVE-2 EU study.^35^ Long-term data from the MOVE-2 Study, Germany,^38^ support these findings. At baseline, 21% of patients had restrictions in several daily activities compared to only 13% reporting such impairments after 12 months. Patients classified as IRs experienced a more prominent improvement.^35,37^

### Safety

#### Adverse events

Data from observational studies have shown that THC:CBD was well tolerated with no new or unexpected side effects emerging. The incidence of AEs varied between 10% and 17% and decreased with prolonged use (Tables 5 and 6). The most common AEs affected the nervous system and comprised dizziness in up to 4%, drowsiness in 1.9%, and fatigue in up to 2.5% of the patients. Nausea was seen in about 2% of the patients (Tables 5 and 6).^31-33,35,37-42,48^ Most of the AEs were mild to moderate and occurred during the titration phase. Likewise, the incidence of AEs of special interest was low (Table 7). Psychiatric or psychotic events were reported in 2.5% of the patients in the international safety registry^31^ and in 6% of the patients in the safety NIS,^41^ and fall-related injuries were described in 6% of patients and 2% of patients had suicidal thoughts or suicide attempts.^31^

**Table 5.** Incidence of adverse events in registries.

	Etges et al^31^	Oreja-Guevara et al^41^	Patti^32^ and Patti et al^33^**
Number of patients in registry, n	941	204	1615
Number of adverse events, n		57	
Number of treatment-related AEs, n (%)		40 (70.2)	
Patients with AEs, n (%)		41 (20)	
Patients with treatment-related AEs, n (%)	123 (13.1)		
Most commonly reported adverse events	Patients with treatment-related AE	Treatment-related AE	Patients discontinue due to treatment-related AE
Nervous system disorder, n (%)	55 (5.8)	15 (26.3)	16 (1.1)
Dizziness	22 (2.3)	3 (5.3)	30 (2.0)
Somnolence	8 (0.9)	2 (3.5)	
Drowsiness	–	–	32 (2.2)
Cognitive effects	–	1 (1.8)	9 (0.6)
Psychiatric disorder, n (%)	27 (2.9)	10 (17.5)	46
Depression	3 (0.3)	3 (5.3)	1 (0.06)
Anxiety	5 (0.5)	1 (1.8)	
Confusion	–	1 (1.8)	
Gastrointestinal disorder, n (%)	32 (3.4)	12 (21.1)	21 (1.4)
Nausea	10 (1.1)	1 (1.8)	
General disorder and administration side conditions, n (%)	26 (2.8)	3 (5.3)	
Oral/mouth/mucosal	–		10 (0.7)
Fatigue	16 (1.7)		36 (2.5)
Serious unrelated AEs, n (%)		8 (3.92)*	5 (0.35)
Serious drug related AEs, n (%)	3 (0.3)	1 (0.5)*	

Abbreviation: AE, adverse event.

* Number of patients; **reporting from the same registry.

**Table 6.** Incidence of adverse events in multi-center NIS.

	Flachenecker w et al^37^	Flachenecker et al long term^38^*	Vermersch and Trojano^35^
Number of patients in NIS, n	325	104	432
Number of adverse events, n	115		
Number of treatment related AEs, n (%)		22	
Patients AEs, n (%)	54 (16.6)		
Patients with drug related AEs, n (%)	51 (15.7)	17 (16.3)	45 (10.4)
Most commonly reported AEs	Patients with AE	Patients with treatment-related AE	Patients with treatment-related AE
Nervous system disorder, n (%)			26 (6.0)
Dizziness	13 (4.0)	1 (1.0)	16 (3.7)
Somnolence			4 (0.9)
Drowsiness	6 (1.9)		
Cognitive effects	–		
Psychiatric disorder, n (%)		4 (3.9)	9 (2.1)
Depression	3 (0.3)		
Anxiety	5 (0.5)	1 (1.0)	1 (0.2)
Confusion	–		
Gastrointestinal disorder, n (%)	32 (3.4)	6 (5.8)	6 ( 1.4)
Nausea	6 (1.9)	1 (1.0)	3 (0.7)
General disorder and administration side conditions, n (%)		4 (3.84)	6 (1.4)
Oral/mouth/mucosal	4 (1.2)	1 (1.0)	1 (0.2)
Fatigue	8 (2.5)	1 (1.0)	3 (0.7)
Serious unrelated AEs, n (%)	8 (2.5		3 (0.7)
Serious drug-related AEs, n (%)	4 (1.2)	1 (1.0)	

Abbreviations: AE, adverse event; NIS, non-interventional study.

* Follow-up of Move 1.^37^

**Table 7.** Incidence of adverse events of special interest.

	Etges et al^31^	Oreja-Guevara et al^41^
Number of patients in registry, n	941	204
Clinically significant AEs, n (%)	216 (23)	
Patients who sought medical attention due to fall-related injury	61 (6)	0
Patients with suicidal thoughts or suicide attempt	15 (2)	0
Other significant psychiatric or psychotic events	55 (6)	5 (2.5)
Change in driving ability		
Improved	63 (7)	5 (2.5)
Deteriorated	19 (2)	1 (0.5)
Both	2 (0.2)	
No change	303 (32)	71 (34.8)
Not recorded	40 (4)	
NA	514 (55)	127 (62.3)

Abbreviations: AE, adverse event; NA, not available.

#### Serious adverse events

In the largest registry,^31^ 113 patients had at least one serious adverse event (SAEs). A total of 24 patients (2.6%) had SAEs that were reported as treatment related and were assigned to the system organ class (SOC) nervous system disorder, psychiatric disorder, or infections and infestations. In the AIFA registry, there were five SAEs (0.3%) namely hypertensive crisis, death after acute myocardial infarction, acute renal failure in a patient with long-term kidney disease, laryngeal carcinoma, and breast cancer.^33^ Eight SAEs were recorded in the Spanish safety study, two of these having a suspected causal relationship with THC:CBD (<1% of sample, ambulation disturbances/polyuria in one and headache in one).^41^ In the NIS, the number of SAEs was low including one fall with fracture,^38^ mental impairment, suicide ideation, and death due to cardiac arrest, all of which were considered to be unrelated to the drug,^35^ and eight further SAEs were reported by Flachenecker et al^37^ and considered related to the medication in four patients (despondency, fatigue, weakness, worsened walking ability, dizziness, muscle spasm, headache, and urinary tract infection).

#### Withdrawal due to adverse events

Etges et al^31^ reported that 25% of the patients had stopped treatment due to AEs. In the Spanish and Italian registry, 14% and 18.7% of the patients, respectively, discontinued THC:CBD secondary to AEs. In the multi-center NIS these rates were 6.3%,^35^ 11.4%^37^ and 7.6%.^38^

#### Driving ability

Driving ability was assessed in the Spanish safety study and international post-marketing risk management safety registry^31,41^ (Table 7). The data from these registries show that most patients reported no impairment of driving ability. On the contrary, 7% of patients in the safety registry and 2.5% of patients in the Spanish safety study rated their driving ability as improved, whereas deteriorations in driving ability were described for 2% and 0.5% of patients, respectively.^31,41^ Driving ability was further investigated in one of the observational studies enrolling 33 new patients with drug-resistant MS spasticity who were treated with THC:CBD as add-on therapy for up to 6 weeks. In this trial, a special test battery (Schuhfried-Wiener Test sytem) was used at baseline and after 4-6 weeks of treatment. At the end of the study, two patients shifted from “unfit” to drive to “fit” and vice versa, while one of the five validated computer-based tests showed statistically significant improvements in favor of Sativex. It was concluded that treatment with THC:CBD did not negatively impact driving ability.^44^

#### Overdose, misuse, abuse, and dependence

Etges et al^31^ reported that 66 patients (7%) in the UK registry had exceeded the maximum recommended daily dose of 12 actuations per day. Around 13-23 sprays were used by 43 patients (4.6%) and more than 24 sprays by 23 patients (2.4%). Of these patients using more than 12 sprays per day, 5 (7.6%) reported AEs (Table 8). Regarding abuse and dependence, a specific questionnaire was completed regarding 392 of 941 patients. The mean duration of THC:CBD exposure was 1091.7 days. Tolerance was reported in two patients (0.5%), but worsening of the condition in these two patients (spasms and pain) was thought to be a possible cause for this finding. In another two patients, evidence of dependence was reported although one of these patients had an incomplete follow-up and for the other there was no proof of abuse, misuse, or psychological dependence.^31^ Collectively, the NIS and registries identified no evidence of abuse, tolerance, or dependence.

**Table 8.** Adverse events in patients with overdose.

Adverse events due to overdose	Sprays/day	Patients, n (%)
Paranoia	15	1 (0.1)
Nausea	16	1 (0.1)
Fatigue	17	1 (0.1)
Falls**	18	1 (0.1)
Anxiety**	30	1 (0.1)

Abbreviation: SAE, serious adverse event.

** Considered drug-related SAE.

## Discussion

Randomized controlled trials are the gold standard generating evidence regarding efficacy and safety. Real-world studies complement efficacy and safety results of RCTs, as they provide data obtained under conditions of routine clinical practice. They can determine whether the expected outcomes are achieved in a larger, more heterogeneous patient population with different co-morbidities not usually included in RCTs and over a longer period of time. In addition, they may address specific clinical questions such as the incidence of special interest AEs.^27,49^ Furthermore, these kinds of studies may gather information on compliance with treatment guidelines, impact on resource use, costs and several other pharmacoeconomic data.

Our systematic review assessed results of real-world studies on THC:CBD spray published since its EU launch in 2011 until September 2017. The 14 papers meeting our inclusion criteria for this review represent a heterogeneous collection of EU real-world data. The data yield could have been expanded by considering conference abstracts. However, we decided to select only peer-reviewed papers, as abstracts—besides the limited availability of information—often present interim data due to the early submission deadlines at conferences. We reviewed data from large registry databases including a safety registry as well as data from international and smaller monocentric retrospective or prospective observational studies.

While all real-world data sources have their limitations, our aim was to present medium to high-quality sources to obtain robust data. The review process of the peer-reviewed journals ensures quality standards of publications. Most of the papers we included reported high-quality data according to our quality criteria (Table 4).

The real-world studies included in this review support the positive benefit-risk balance during long-term use of THC:CBD spray in everyday practice. The findings of our review are generally in line with the results of the RCTs.^21–24^ There were no differences in the baseline characteristics of patients in the real-world studies compared to the RCTs with an average age of 50 years and a gender distribution toward slightly more female patients.^21-24^

Most of the included studies applied the enriched study design first used in the RCT by Novotna et al^23^ to identify IRs who had at least a 20% reduction in mean NRS spasticity score within the first month of treatment with THC:CBD. The NRS is a valid and reliable patient-reported outcome measure to assess MS spasticity. It has been shown that 20% reduction of the mean NRS is the minimal change which would be classified as clinically relevant (minimal clinical important difference, MCID). After 4 weeks, 47.5% resp. 70% of patients in the above mentioned RCT reached the threshold of the MCID referred to as IRs.^23,24^ The proportion of IRs in the real-world studies ranged from 41.9%^37^ in the German MOVE-2 Study to 82.9%^36^ of patients MOVE-2 Italian interim analysis on Italian patients. The Italian AIFA registry reported an initial response rate of around 70%^32,33^ which was confirmed by another recent Italian observational study report an initial response of 71.7%.^39^ The higher initial response rate may partly be due to stricter inclusion criteria for Italian patients entered in the compulsory web-based registry not allowing entry if the baseline NRS for spasticity is below 4 as well as the automatic calculation within a strict time frame after therapy initiation.^33^ Higher response rates may also be achieved in clinical settings, where experienced clinicians ensure proper dose titration, adequate dosing, and correct use of the spray.

NRS spasticity score was the main outcome measure in the Italian registry as well as in most of the observational studies which aimed to evaluate clinical effectiveness. In addition, few studies also measured spasticity using the MAS. The results of the observational studies are in line with the RCT results showing a reduction in the NRS over time achieved with THC:CBD. In addition, spasticity-associated symptoms, that is, spasm counts, pain, and sleep impairment improved during the study period.^35,37^

The average daily dose in the observational studies was five to six sprays. In comparison, RCTs established a higher average daily dose of eight sprays. In a recently published RCT, patients used the opportunity to adjust their daily dose during the entire study period, and the average daily dose was seven sprays per day.^24^ This suggests that clinical effectiveness can be achieved and also maintained with lower doses of THC:CBD in the routine clinical setting. Furthermore, this has shown that the advantage of the THC:CBD oromucosal spray of being able to individually adjust the dosage depending on efficacy and tolerability is used by patients.

THC:CBD was well tolerated in the evaluated studies in the same way as in the RCTs. No new safety signals emerged in the real-world setting. This review contributes to the safety data already collected in RCTs as it analyses data from longer running studies and specific safety registries on THC:CBD. The most frequent AEs were mild-to-moderate transient dizziness and fatigue (Table 5). Other safety outcomes such as abuse and tolerance, which will be detected only in long-term follow-up and large-sample studies, have not been observed in the reviewed studies. Only 5 of 43 patients who took a higher-than-recommended dose (up to 30 actuations of THC:CBD per day) reported AEs which were considered drug related, with two of these five patients experiencing SAEs. All other patients who took higher than recommended doses tolerated these well. Likewise, the evaluation of AEs of special interest did not reveal any new and unexpected safety concerns.^31^ Furthermore, no safety risk of driving impairment has emerged from the real-world studies.^31,41,44^

Besides the advantages of gaining complementary evidence from real-world data, there are some limitations in compiling and evaluating these data sources. These include a reporting bias, that is, selective reporting of results leading to a possible overestimation of the efficacy and under-estimation of safety aspects. Other limitations arise from imprecise definitions of outcome criteria or only partial collection of established outcome criteria, non-harmonized data collection, incomplete follow-up data, and/or lack of information on how missing data was handled. In addition, the study population could be too heterogeneous to transfer the results to the whole patient population. Nevertheless, as mentioned before, this kind of data is essential for the benefit–risk assessment of a medicinal product in clinical practice. Therefore, further development of new standardized approaches to overcome limitations of real-world data is prerequisite.^46^

## Conclusions

The data evaluated in this systematic literature review provide evidence for the efficacy and safety of THC:CBD in real-world clinical practice. They confirm the results obtained in RCTs. In therapy-resistant spasticity, that is, in patients not adequately responding to or not tolerating previous anti-spastic drugs, the add-on use of THC:CBD is an effective therapeutic option with a good tolerability and safety profile. No new or unexpected AEs have been reported in clinical practice, and there are no indications of abuse or tolerance development with long-term use. As treatment in the real-world setting has shown, one of the great advantages of this THC:CBD formulation is that responders can easily be recognized during the first 4 weeks of treatment, and the dosage can be individually titrated depending on the patient’s needs. In summary, these data illustrate that THC:CBD is an effective and tolerable alternative therapeutic option for patients who do not respond to conventional anti-spastic drugs and still suffer from MS spasticity.

## Appendix 1

**Table A1.** Electronic search terms used in database searches.

#	Searches
Medline	
1	“thc AND cbd”
2	“Delta 9 Tetrahydrocannabinol AND Cannabidiol”
3	“Sativex”
4	“Nabiximols”
5	“gw 1000”
6	“Cannabis” AND “Extract”
7	Cannabinoid*
8	#1 OR #2 OR #3 OR #4 OR #5 OR #6 OR #7
9	“Multiple Sclerosis”
10	Spastic*
11	(“real world”) OR “real life”
12	regist*
13	observation*
14	“non-interventional”
15	“longitudinal”
16	(“retrospective”) OR “prospective”
17	Database
18	#11 OR #12 OR #13 OR #14 OR #15 OR #16 OR #17
19	#8 AND #9 AND #10 AND #18
Embase	
1	((thc AND cbd) OR (“Delta 9 Tetrahydrocannabinol” AND “Cannabidiol”) OR Sativex OR Nabiximols OR (Cannabis AND Extract) OR Cannabinoid*)
2	AND (“Multiple Sclerosis”)
3	AND (Spastic*)
4	AND ((“real world”) OR (“real life”) OR regist* OR observation* OR (“non-interventional”) OR longitudinal OR retrospective OR prospective OR Database)
Cochrane library	
1	“Multiple Sclerosis” AND Spastic* AND Cannabi*
